# Narrative review of patient-specific 3D visualization and reality technologies in skull base neurosurgery: enhancements in surgical training, planning, and navigation

**DOI:** 10.3389/fsurg.2024.1427844

**Published:** 2024-07-16

**Authors:** Ilkay Isikay, Efecan Cekic, Baylar Baylarov, Osman Tunc, Sahin Hanalioglu

**Affiliations:** ^1^ Department of Neurosurgery, Faculty of Medicine, Hacettepe University, Ankara, Türkiye; ^2^ Neurosurgery Clinic, Polatli Duatepe State Hospital, Ankara, Türkiye; ^3^ Btech Innovation, METU Technopark, Ankara, Türkiye

**Keywords:** augmented (virtual) reality, mixed reality, 3D printing, 3D model, skull base, neurosurgery

## Abstract

Recent advances in medical imaging, computer vision, 3-dimensional (3D) modeling, and artificial intelligence (AI) integrated technologies paved the way for generating patient-specific, realistic 3D visualization of pathological anatomy in neurosurgical conditions. Immersive surgical simulations through augmented reality (AR), virtual reality (VR), mixed reality (MxR), extended reality (XR), and 3D printing applications further increased their utilization in current surgical practice and training. This narrative review investigates state-of-the-art studies, the limitations of these technologies, and future directions for them in the field of skull base surgery. We begin with a methodology summary to create accurate 3D models customized for each patient by combining several imaging modalities. Then, we explore how these models are employed in surgical planning simulations and real-time navigation systems in surgical procedures involving the anterior, middle, and posterior cranial skull bases, including endoscopic and open microsurgical operations. We also evaluate their influence on surgical decision-making, performance, and education. Accumulating evidence demonstrates that these technologies can enhance the visibility of the neuroanatomical structures situated at the cranial base and assist surgeons in preoperative planning and intraoperative navigation, thus showing great potential to improve surgical results and reduce complications. Maximum effectiveness can be achieved in approach selection, patient positioning, craniotomy placement, anti-target avoidance, and comprehension of spatial interrelationships of neurovascular structures. Finally, we present the obstacles and possible future paths for the broader implementation of these groundbreaking methods in neurosurgery, highlighting the importance of ongoing technological advancements and interdisciplinary collaboration to improve the accuracy and usefulness of 3D visualization and reality technologies in skull base surgeries.

## Introduction

The skull base represents one of the most complex areas in human anatomy, comprising important neurovascular structures within an intricate space (1). The skull base is anatomically divided into anterior, middle, and posterior regions. While some neurosurgical pathologies stay in one, many extend beyond the borders of a particular cranial fossa. For instance, in the skull's center, sellar/parasellar tumors can extend to all three cranial fossae. Endoscopic and open (transcranial) approaches can be utilized separately or in combination to tackle these complex pathologies. Understanding its structure and performing neurosurgical operations requires experience that requires visuospatial orientation with accuracy (2, 3). Skull base surgery's inherent challenges, characterized by complex neuroanatomy, proximity of critical structures, and limited surgical access, underscore the need for highly advanced technologies that enhance the surgeon's capabilities beyond conventional limits (4, 5).

To solve these difficulties, personalized three-dimensional (3D) visualization and reality technologies have become essential in planning surgeries and guiding neurosurgeons during operations (6–8). The synthesis of these 3D technologies entails the utilization of sophisticated software to handle radiography data, hence facilitating the creation of anatomically precise 3D digital and printed models (9, 10). These tangible models serve as a physical navigational map, allowing surgeons to visualize and strategize operations with comprehensive and unprecedented clarity (11–13) A comprehensive knowledge of the specific examples of skull base disorders is required to create these models, encompassing the entire process from 2D image acquisition to the final 3D anatomic and/or pathologic visualization (14–16).

The advancement of imaging and modeling approaches, including augmented reality (AR), virtual reality (VR), extended reality (XR), mixed reality (MxR), and 3D printing, has shown inventive solutions that cater to the distinct requirements of each area (17–19) AR overlays digital information, such as MR images, onto the actual scenario, enhancing the surgeon's view during procedures (20). VR immerses users in a virtual environment, allowing detailed preoperative planning and surgical rehearsals (21). MxR merges real and virtual worlds, enabling interaction with physical and digital objects in real-time, which is crucial for navigating complex skull base surgeries (22). XR encompasses AR, VR, and mixed reality, offering diverse immersive experiences (23). These technologies improve the surgeon's ability to see, allowing them to perceive and interact with virtual preoperative planning, as demonstrated by studies (24–26) These tools can offer real-time, intraoperative navigation during the procedure, enhancing the surgeon's understanding, potentially decreasing surgical complications, and enhancing patient outcomes (27, 28).

Our study is a narrative review of patient-specific 3D visualization and reality technologies in skull base surgery. We delicately evaluated and synthesized the existing literature on this topic. We investigated the role of these technologies in the surgical treatment of skull base diseases. This study aimed to enhance the profession by synthesizing previous studies and providing guidance to specialists on the intricate anatomy of the skull base. Another objective was to assess the progress of endoscopic and open surgery techniques using AR, VR, XR, MxR, and 3D printing advancements in each specific area of the skull base. We also evaluated how these innovations impact the surgeon's approach, decision-making, and surgical performance. Our overall aim was to create a thorough resource that explains the pros and cons of these technologies and how they can be incorporated into future innovations related to the skull base.

## Generating 3D digital and printing models for neurosurgical planning and navigation

### Radiological data acquisition and segmentation

For building 3D visualization models for neurosurgical planning, the acquisition and segmentation of radiological 2D images play a pivotal role. Magnetic Resonance Imaging (MRI) is generally preferred for its superior soft tissue contrast, which facilitates the delineation of neural tissues. At the same time, Computerized Tomography (CT) scans provide details of bony structures (29, 30). Detailed T1-weighted MRIs catch the skin's topography, creating a surface map for external anatomical points. CT angiography defines the skull and vascular network, providing structural clarity and critical detail on bone and vessels. White and grey matter borders are extracted from T1W and T2W MRI sequences, allowing for the differentiation of cerebral tissue layers. Veins and sinuses are delineated with contrast-enhanced MR venography, providing insight into the venous system. At the same time, arterial structures are highlighted using time-of-flight (TOF) MR angiography, focusing on blood flow dynamics. Pathological cases such as tumors and associated edematous changes are identified through contrast-enhanced MRI sequences, including T1W, T2W, and Fluid attenuated inversion recovery (FLAIR). After alignment and calibration, this multimodal imaging synthesis produces a 3D visualization reflecting the true anatomical complexity essential for skull base operations (8, 31).

Advanced software tools (e.g., Materialise Mimics) are then used to segment anatomic structures on radiological images. This stage is pivotal as it differentiates between different anatomical features by applying thresholds that recognize variations in tissue density and radiological characteristics using the abovementioned modalities. Segmenting anatomical datasets requires both automated algorithms and manual refinement to ensure precision, which might involve expert radiologists and neurosurgeons for verification (32, 33).

### 3D reconstruction and model refinement

3D reconstruction follows segmentation, typically through surface or volume rendering techniques. These methods convert the segmented 2D slices into digital 3D models, which then undergo refinement. Available software tools (e.g., Materialise 3-matic) allow for smoothing, optimizing mesh structures, and making anatomical adjustments to ensure the digital model's alignment with the original anatomy (31, 34, 35).

### Integration into AR/VR/MxR/XR environments

For AR/VR applications, the 3D models are imported into software environments. Systems like Unity or Unreal Engine can create interactive virtual surgical anatomy, pathology, and surgical operations. The integration usually includes programming for interactions with the model, such as simulating surgical interventions and AR display during the surgeries (36, 37). For AR and MxR, the models are processed through platforms like Microsoft's HoloLens®, facilitating their overlay onto real situations or into an MxR environment. This step is crucial for surgical planning, rehearsal, education, or intraoperative guidance, especially in skull base surgery (38–40).

### 3D printing protocols

For tangible models, the 3D files are prepared for 3D printing via slicing software, translating the model into a series of cross-sectional layers. Parameters such as layer thickness, orientation, and support structures are optimized based on the selected 3D printing technology, such as Stereolithography (SLA), Fused Deposition Modeling (FDM), or Selective Laser Sintering (SLS) (41, 42). Post-processing steps such as removing supports, surface smoothing, and sterilization are essential, especially for models used for surgical purposes (11, 43).

### Quality assurance and clinical validation

Collaboration between clinicians, radiologists, and biomedical engineers is required to validate the production of clinically relevant and accurate models. Verification of the models is a very important phase in which the 3D structures are compared with the original imaging to verify accuracy (44, 45). This can be done using analytical software tools that quantify deviations between the models and the radiological source. Clinical validation may also involve using the models in surgical settings or via comparison with intraoperative findings to assess their practical utility and reliability by the surgeons during operations (31).

## Anterior skull base surgery

The anterior skull base is situated in the region that lies between the cranial and facial compartments. The object can be divided into three clearly defined regions: the midline and two lateral side regions. The midline segment comprises the cribriform plate, posterior frontal plate, ethmoidal roof, planum sphenoidale, and tuberculum sellae. The roof of the nasal cavity serves as a crucial barrier between the sinonasal tract and the intracranial space. The boundaries between the intracranial compartment and the orbital contents are primarily defined by the lateral segments, which consist principally of the orbital plates of the frontal bones and the smaller wings of the sphenoid. In addition, the midline limbus sphenoidale also contributes to this delineation (46, 47).

This design not only maintains the skull's structural strength but also exposes potential weaknesses for different diseases that may penetrate the front part of the cranial cavity. This region can exhibit benign tumors, such as meningiomas, and malignancies, such as squamous cell carcinomas and esthesioneuroblastomas (48). These disorders frequently exploit the skull base's inherent pathways and delicate barriers, resulting in potential difficulties such as cerebrospinal fluid leaks and the spread of the ailment within the skull (49, 50).

### Endoscopic approaches

**Figure 1:**
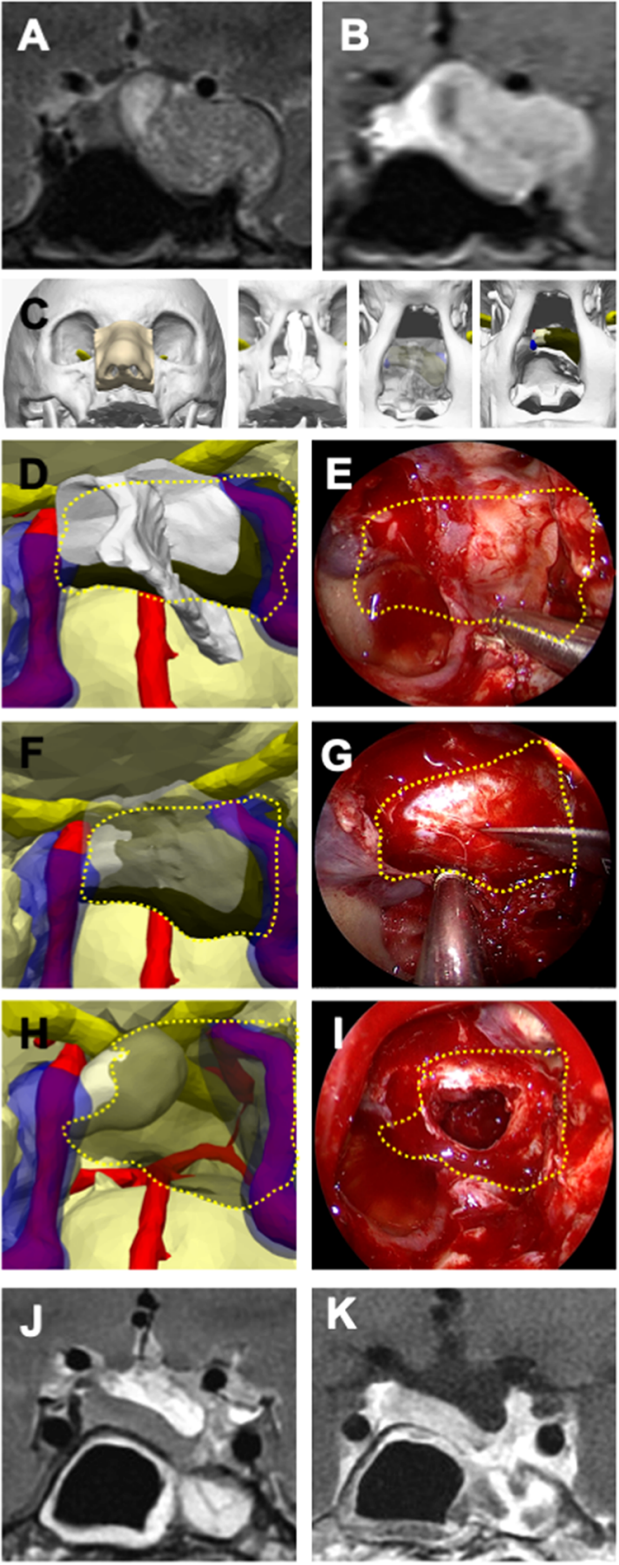
Illustrative case 1—pituitary adenoma in a 40-year-old female patient. (**A**) Preoperative T2-weighted and (**B**) T1-weighted MRI with gadolinium contrast revealed a pituitary adenoma located in the left cavernous sinus and left temporal fossa on coronal sections. (**C**) A 3D digital model illustrates the stepwise surgical anatomy for the nasal and sphenoidal stages of endoscopic endonasal transsphenoidal surgery, extending from the nostril to the sphenoid sinus and sellar floor. (**D**) The bone structures were rendered transparent on the 3D model, and a plan for creating an opening in the sellar floor was devised, taking into account the intersphenoidal septum, the bilateral internal carotid arteries (ICA), and the cavernous sinus. (**E**) The tumor borders are delineated with a dashed yellow line, as similarly depicted on the endoscopic image. (**F**) The sellar floor was digitally removed in the 3D model, the optimal site for the dural incision was determined, and (**G**) this plan was executed during endoscopic surgery. The tumor borders are indicated by a dashed yellow line. (**H**) The 3D model displays the anatomical relationship of the tumor with the normal pituitary gland, the ICA, and the cavernous sinuses, (**I**) illustrating its removal from the inside of the sella and the left cavernous sinus. (**J**) Postoperative T2-weighted and (**K**) T1-weighted MRI coronal sections with gadolinium contrast have shown millimetric residues.

The endoscopic endonasal approach (EEA) for anterior skull base surgery has been significantly advanced by 3D modeling (Figure 1) and AR/MxR/XR (7, 51). These technologies have been transformative in treating pathologies such as complex meningiomas, chordomas, chondrosarcomas, and sinonasal malignancies that extend into the anterior skull base (5, 52). Developing 3D models from patient-specific imaging data has further transformed surgical planning, and there has been increasing interest in neurosurgery, as summarized in Table 1. 3D models have provided surgeons with a malleable visualization of the target pathology close to the intricate intracranial structures (70). These reconstructions act as a real-time navigational guide, markedly reducing the risk to critical neurovascular structures (75, 76). Moreover, incorporating AR technology into the operational field provides surgeons with a virtual overlay that enhances their 3D orientation, enabling precise tumor excision while maintaining antitarget avoidance (77). Consequently, using these technologies has demonstrated measurable improvements in surgical outcomes. For instance, the adoption of AR and 3D visualization has been associated with a reduction in cerebrospinal fluid (CSF) leak rates from 40% to as low as 2.9% in more recent series, a decrease in cranial nerve (CN) dysfunction rates, and a reduction in internal carotid artery (ICA) injury rates from 0.9% to 0.3%, thereby highlighting the potential of these technologies to significantly enhance surgical precision and patient safety (59–79).

**Table 1:** Advancing skull base neurosurgery: A review of 3D innovations in surgical planning and guidance.

Author	Article type	Technique	Hardware	Software	Neurosurgical approach	Key findings
Jean (53)	Case Series	Mixed reality in cranial surgery	Preoperative CT and MRI, Microscope-integrated AR	Surgical Theater SRP and SyncAR, StealthStation S8	Various skull base approaches	MxR facilitates surgical planning and execution,with a learning curve but no surgery or hospitalization extension.
Gómez Amarillo et al. (19)	Mini-Review	AR for intracranial meningioma resection	Microscopes with integrated HUDs, HMDs	AR platforms, 3D reconstruction software	Meningioma resection, especially skull base	AR enhances surgery by improving visualization of critical structures and tumor boundaries.
Yamaoka et al. (28)	Research Article	3D printing	Multi-detector row computed tomography data, 3D printer	N/A	Epidural procedures, skull base drilling, dural peeling techniques	The 3D model of the anterior and middle cranial fossa is an effective tool for teaching anatomical knowledge and essential skull base surgery skills, including dural dissection and 3D positioning and presurgical planning of structures.
Salgado-Lopez et al. (54)	Video Article	Intraoperative heads-up display	Endoscope, doppler, heads-up display equipment	Virtual reality integration software smartbrush	Anterior skull base surgery via pterional craniotomy	Implementation of virtual reality and heads-up display in skull base surgery for enhanced visualization and navigation, ensuring artery preservation and tumor resection.
Jean et al. (39)	2D Operative Video	AR/VR	Navigation-tracked microscope	Augmented reality (AR) template, Virtual reality (VR) rendering	Resection of clinoid meningioma	AR and VR enhance surgical planning and execution, ensuring precise and minimally invasive approaches.
Zawy Alsofy et al. (6)	Research Article	VR	CT and MRI scans	3D slicer, VR software	Anterior skull base meningioma resection	3D-VR significantly influences the detection of tumor-related anatomical structures, recommended head positioning, and surgical approach.
Zeiger, et al. (55)	Retrospective Study	Use of 3D digital reconstructions and mixed reality for intraoperative navigation for EEA	Surgical Theater's 3D reconstructions, Brainlab's Cranial Navigation system	DICOM-based 3D reconstruction software from Surgical Theater, Brainlab's optical tracking software	Endoscopic endonasal skull base surgery	Mixed reality technology improved spatial awareness and operative efficiency in skull base surgery.
Lai, et al. (56)	Original Article	Augmented reality (AR) with fusion of intraoperative CBCT on endoscopic view	Endoscope with camera, C-arm for CBCT, OTS with video cameras	ARSN system, optical tracking	Endoscopic endonasal skull base surgery	Novel AR technique integrating endoscopic and intraoperative CBCT imaging showed sub-millimeter accuracy, potentially increasing safety and efficiency in endoscopic skull base surgery.
Citardi et al. (57)	Literature Review	Augmented reality, microsensors	Compact navigation systems, EM tracking	Preoperative Planning Software, AR-Enhanced Navigation	Endoscopic sinus and anterior skull base surgery	Advanced surgical navigation aims for TRE of 1.0 to 1.5 mm. The incorporation of AR technology and microsensors may significantly enhance precision and safety in sinus and skull base surgery.
Li et al. (58)	Feasibility Study	AR navigation system	Endoscopy imaging system, infrared tracking system, workstation	Custom open-source software	Endoscopic sinus and skull base surgery	The AR navigation system provides enhanced visual guidance, reducing operation time and mental workload, especially beneficial for less experienced surgeons.
Porras et al. (59)	Systematic Review	Endoscopic endonasal approach (EEA)	Endoscopy and intraoperative navigation systems.	Image-guided surgery software, electronic medical records for data analysis, and possibly software for modeling and simulations in surgical planning.	EEA to the skull base, addressing pathologies in anterior, middle, and posterior cranial fossae.	The review highlights the advantages of EEA, including a direct trajectory to ventral skull base lesions, avoidance of brain retraction, and improved visualization. The authors stress the importance of understanding and preventing complications such as CSF leaks, cranial nerve dysfunction, pituitary gland dysfunction, ICA injury, infection, and other potential issues.
Lai et al. (60)	Research Article	High-fidelity virtual reality simulation	Microcomputed tomography scans	CardinalSim software	Middle cranial skull base approaches	3D simulations illustrate neurovascular relationships and interactive drilling in the middle cranial fossa for training, education, and surgical planning purposes.
Thavarajasingam et al. (52)	Systematic Review	AR in transsphenoidal surgery	Various, including optical tracking systems and hybrid endoscopic-AR displays	Various, including ITK-SNAP 2.0, Scopis, Brainlab, etc.	Transsphenoidal endoscopic endonasal surgery (ETS) and microscopic transsphenoidal surgery (MTS)	AR enhances landmark identification and intraoperative navigation, positively impacting surgeon experience and potentially improving accuracy and surgical time. However, the impact on patient outcomes is unclear.
Jean et al. (38)	Case Report	Augmented reality	AR navigation-tracked microscope	Virtual reality rendering software	Anterior petrosectomy	AR provides critical visual cues during AP, enhancing safety and protecting neurovascular structures.
Guo et al. (9)	Retrospective Study	3D-printed models for surgery	CT, MRI, and CT angiography	Mimics software	Skull base meningioma surgery	3D-printed models significantly aid in surgical planning, anatomical understanding, and patient education for skull base meningiomas.
Jean and Singh (61)	2D Operative Video	VR	Endoscope,VR	Virtual reality (VR) rendering	Endoscopic endonasal approach for tuberculum sellae meningioma	Preoperative planning and surgical rehearsal in VR can improve the efficiency of endoscopic skull base surgery.
Lee and Wong (18)	Literature Review	AR/VR	Dextroscope VR System, ImmersiveTouch, NeuroTouch Simulator	NeuroPlanner, NeuroBase, Dextroscope VIVIAN	Management of intracranial tumors, specifically focusing on skull base tumor surgeries	VR and AR technologies are instrumental in surgical planning, providing 3D visualization of anatomical structures and facilitating the precise excision of skull base tumors.
Carl, et al. (62)	Original Article	Microscope-based AR for visualization of the target and risk structures in transsphenoidal surgery	Operating microscopes with integrated head-up displays, intraoperative computed tomography (iCT)	AR visualization, automatic registration using iCT	Transsphenoidal surgery	Microscope-based AR significantly increased accuracy and safety in complex transsphenoidal procedures. Automatic iCT-based registration provided high precision, suggesting it is a reliable tool for enhancing patient safety.
Barber et al. (11)	Case Study	AR, surgical navigation, and 3D printing	CT imaging, Form2 3D printer, Stealth3D workstation	ITK-SNAP, Unity with Vuforia, Android OS	Transcanal endoscopic approach to the petrous apex	AR and 3D-printed patient-specific models coupled with navigation for preoperative planning provided a realistic simulation for complex lateral skull base surgery, which closely mirrored intraoperative findings and may improve surgical outcomes.
Sato et al. (27)	Research Article	3D-MFI	High-Resolution MRI—Computed Tomography (CT)—Digital Subtraction Angiography (DSA)	Avizo software (version 6.0, Visage Imaging, CA)	Surgical simulation for resection of deep-seated meningiomas	3D-MFI is effective for surgical planning and education, with precise identification of skull base structures and vessels.
McJunkin, et al. (63)	Research Article	Mixed reality platform development	MR head mounted display (HMD), Microsoft HoloLens	Unity® gaming platform, Visual Studio, ITK-SNAP, MeshLab	Lateral skull base approach	The MR platform effectively visualized temporal bone structures. It improved spatial understanding of anatomy, potentially enhancing surgical navigation and training.
Randazzo et al. (14)	Systematic Review	3D printing for surgical planning	CT, MRI	ITK-SNAP, Unity, Vuforia	Transcanal endoscopic approach to the petrous apex	3D printing and AR facilitate virtual surgical planning and simulation, potentially improving patient safety and surgical outcomes in skull base neurosurgery.
Kawamata et al. (64)	Technical Note	Endoscopic augmented reality navigation	Rigid endoscope with LEDs, optical tracking system	N/A	Endonasal transsphenoidal surgery	AR system improves tumor and anatomic structure visualization during surgery, enhancing safety and accuracy for pituitary tumor treatment.
Olexa, et al. (65)	Case Report	Augmented reality (AR) visualization and planning	Microsoft Hololens 2 Head-Mounted Display	Custom application developed by hoth intelligence (Philadelphia, Pennsylvania)	Retrosigmoid craniotomy for vestibular schwannoma	AR technology streamlined surgical planning for vestibular schwannoma, allowing for accurate 3D mapping and visualization of the patient's head, aiding retrosigmoid craniotomy approach decisions.
Hong et al. (66)	Research Study	Augmented reality neuronavigation	Mobile-based system	Self-developed mobile augmented reality navigation system (MARNS)	Retrosigmoid craniotomy	MARNS accurately located the transverse-sigmoid sinus junction with a mean error of 2.88 mm and an average positioning time of 279.71 s. It maintained bone flap integrity in all cases, providing a convenient, cost-effective, and reliable method for neurosurgical navigation.
Schwam et al. (67)	Preliminary Report	Augmented reality in posterolateral skull base surgery	BrainLab Curve™, Surgical Theater, Zeiss OPMI® PENTERO® 900 Microscope	N/A	Posterolateral skull base surgery, specifically for cerebellopontine angle tumor resection	AR showed utility in the preparatory and approach phases of posterolateral skull base surgery, particularly in simulating tumor resection, planning incisions, and craniotomies.
Martín-Noguerol et al. (17)	Literature Review	Hybrid CT & MRI 3D printing	Multimodality 3D printers	Registration and segmentation software	Skull base, CNS, spinal surgery planning	Hybrid models enhance pre-operative planning and surgical precision, especially for complex skull base neurosurgeries.
Lin et al. (68)	Original Article	3D Printing of Cranial Nerves	Connex3 Objet350 3D printer	Mimics Research v17.0, 3-matic v9.0	Transnasal, frontotemporal, and retrosigmoid approaches	3D printed models enhanced the visualization of skull base structures and cranial nerves, facilitated surgical simulation and planning, and improved the accuracy of cranial nerve reconstruction during surgery.
Mascitelli, et al. (69)	Clinical Study	HUD/augmented reality	Brain Lab curve™, Zeiss Pentero 900	Brainlab navigation, surgical theater	Multimodal, intracranial surgery for skull base and vascular cases	HUD is effective for a range of vascular and oncologic intracranial pathologies in skull base surgery, aiding in various stages from skin incision to arachnoid dissection. Excellent or good accuracy was achieved in most cases; deep lesions had less accuracy. No HUD-related complications were reported.
Pacione et al. (10)	Technical Note	Multimaterial 3D printing	Dual-energy CT, somatom force scanner, objet260 dental selection 3D printer	Syngo.via, Intellispace Portal, STL file format	Complex deformity of skull base and craniovertebral junction surgery	3D-printed models enhanced surgical planning for complex deformities and were instrumental in choosing the most effective approach and correction strategy.
Baskaran et al. (16)	Literature Review	3D printing and rapid prototyping	Various 3D printers using powder-based materials such as polymers, ceramics, plastics, resins, super alloys, stainless steel, titanium	DICOM images and converting them to STL file format such as Freesurfer and InVesalius, as well as 3D modeling software like Blender	Anatomical modeling, skull base training	3D printing is precise for neurosurgical planning, especially for complex areas like the skull base. It enhances anatomical education and pre-operative simulation.
Dixon et al. (70)	Research Article	LIVE-IGS, critical structure proximity alerts, 3D virtual endoscopy	Optical IGS reflective markers, endoscope, drill, CBCT system	ITK-SNAP 2.0, custom navigation software	Endoscopic transclival approaches	The LIVE-IGS system reduced mental demand, effort, and frustration compared to conventional IGS. It provided accurate, intuitive, and dynamic feedback, which could improve spatial awareness and reduce task workload during surgery.
Cabrilo et al. (71)	Technical Note	Augmented reality-assisted neuronavigation	Operating microscope, neuronavigation workstation	BrainLAB's Iplan platform	Skull base surgery for clivus chordoma	Augmented reality-based neuronavigation overlays 3D neuronavigational data onto the operating field, improving navigation throughout skull base procedures and offering a more intuitive form of image-guided surgery without additional hardware.
Oishi et al. (72)	Research Article	3D imaging and modeling, interactive virtual simulation (IVS)	3D printer for plaster models, haptic device	CAD software with 3D Capabilities	Skull base and deep tumor surgery	3D imaging and modeling enhanced understanding of complex anatomical relationships, provided realistic simulation for surgical planning and training, and allowed for the determination of optimal surgical strategies for skull base and deep tumors.
Oishi et al. (73)	Research Article	3D multifusion volumetric imaging (3D MFVI), including volume rendering and image fusion	64-channel multislice CT scanner, 1.5-T MRI imaging system, DSA system	Image-analysis software (Real Intage; KGT, Inc)	Various approaches based on tumor location and type (e.g., retrosigmoid approach with IAC exposure, extended transsphenoidal, etc.)	3D MFVI techniques enabled adequate visualization of the microsurgical anatomy, facilitated presurgical simulation, and allowed surgeons to determine an appropriate and feasible surgical approach.
Rosahl et al. (74)	Original Article	VR augmentation	Infrared-based image-guidance system	Image guidance laboratories software	Various skull base procedures	VR augmentation can enhance skull base surgery by providing a déjà vu experience of the surgical field, eliminating the need for mental reconstruction of 2D images and potentially improving surgical outcomes.

In a video article, the authors studied the “Expanded Endoscopic Endonasal Transtuberculum Approach” for resecting a tuberculum sellae meningioma, utilizing the Surgical Theater SRP7.4.0 (Cleveland, Ohio) for VR preoperative planning and surgical rehearsal. This approach was applied to a 57-year-old female patient presenting with sudden right abducens palsy. The VR simulation demonstrated the absence of anterior cerebral artery enclosure, the tumor's non-extension beyond the ICA laterally, and the adequacy of the surgical corridor for the endonasal approach. Despite lacking haptic feedback, the VR rehearsal significantly contributed to the procedural planning by allowing a 360-degree, multicolored, 3D visualization of the tumor. This method underscored the potential of VR in enhancing surgical precision and efficiency, particularly for surgeons in the early stages of their endoscopic career (61).

In recent studies into the EEA in skull base surgery, researchers have employed a novel platform by Surgical Theater® (55, 61). This platform amalgamates high-definition preoperative imaging data to construct detailed 3D patient anatomy models. This virtual model is then integrated into intraoperative navigation systems, which employ optical tracking to align the virtual and physical surgical fields. The synergy of Surgical Theater's 3D reconstructions with navigation technology provides an MxR view that assists surgeons in real-time during surgery. In a recent study, the authors explored MxR visualization in EEA, focusing on a novel technology that combines 3D reconstructions of patient anatomy with intraoperative navigation. Analyzing 134 retrospective cases, MxR facilitated the surgical approach by identifying critical anatomical structures such as the internal carotid arteries (ICA) and optic nerves, improving the safety and efficiency of the procedures. This initial experience suggests that MxR visualization is valuable in complex skull base surgeries (55).

The integration of AR using operating microscopes equipped with head-up displays has been transformative. Surgeons can perform automatic registration by utilizing intraoperative computed tomography (iCT), thus significantly enhancing navigational accuracy. This precision is especially beneficial in transsphenoidal surgeries for anterior skull base tumors, where AR aids in the differentiation of tumor margins and in avoiding critical neurovascular structures like ICA and cranial nerves. The resulting decrease in target registration error (TRE) has been influential in reducing the risk of vascular injury and ensuring near or gross total tumor resection (54, 69).

In a study published in 2019, the authors studied the application of AR in transsphenoidal surgery through microscope-based head-up displays. The study, encompassing 288 transsphenoidal procedures by a single surgeon, integrated AR for 47 patients (16.3%), highlighting its incorporation into the surgical field. The AR's accuracy is related to navigation and microscope calibration, with fiducial-based registration yielding a TRE of 2.33 ± 1.30 mm. Automatic registration using iCT highly improved AR accuracy to a TRE of 0.83 ± 0.44 mm (*P* < 0.001). Additionally, using low-dose iCT protocols minimized radiation exposure to the level of a single chest radiograph (0.041 mSv). This advancement in AR technology provides increased patient safety in complex procedures by significantly enhancing the accuracy of intraoperative navigation, reducing radiation exposure, and facilitating better orientation for the surgeon. Notably, no vascular injuries or new neurological deficits were reported in 47 AR-assisted transsphenoidal procedures, indicating that AR enhances surgical orientation and comfort, thus contributing to patient safety. The significant reduction in TRE ensures accurate alignment of the AR overlay with the patient's anatomy, minimizing the risk of surgical errors. The low-dose iCT protocols contribute to patient safety by substantially reducing radiation exposure. These findings emphasize the potential of AR in endoscopic skull base surgery, making procedures safer and more efficient (62).

Another study evaluated the incorporation of AR imaging with the endoscopic view in endonasal skull base surgery. This surgical navigation technique demonstrated sub-millimeter accuracy, employing augmented reality surgical navigation systems (ARSN) with 3D cone beam computed tomography (CBCT). The study verified the accuracy of CBCT image co-registration on the endoscopic view, with a mean TRE of 0.55 mm and a standard deviation of 0.24 mm. This approach ensures precise surgical navigation and offers real-time endoscopic and diagnostic imaging (56).

AR/MxR/XR and microsensors offer surgeons unparalleled visualization and instrument-tracking capabilities. Integrating AR with surgical navigation systems merges preoperative imaging data with real-time surgical views, providing a more intuitive surgical experience and potentially reducing complications. Microsensors, especially in electromagnetic tracking systems, enable the placement of instruments and targeted therapies, even in the challenging anatomical landscapes of the sinus and skull base. These advancements signify achieving surgical navigation with sub-millimeter accuracy and increase the possibilities of minimally invasive surgical techniques. Additionally, microsensors have been shown to offer advantages over conventional neuronavigation and stereotaxis. For example, Citardi et al. reported that while traditional systems often achieve a TRE of 1.5–2.0 mm, including microsensors can reduce this to below 1 mm. In practical terms, these improvements in accuracy can significantly impact surgical outcomes. For instance, the enhanced precision afforded by microsensors has been associated with reduced intraoperative blood loss and fewer major and minor complications. Studies have demonstrated that surgeries utilizing these navigation systems experience a 25% rate of intraoperative adjustments based on real-time feedback. This has led to better surgical outcomes and reduced the need for revisions. These findings support the enhanced safety and efficacy of using microsensors in surgical navigation, justifying their adoption for surgeries (57).

In another study, the authors integrated AR with surgical navigation in the cadaver study, demonstrating its potential for endoscopic sinus surgery. The study utilized the Scopis Hybrid Navigation system to overlay preoperative CT images onto real-time surgical views, achieving accuracy better than 1.5 mm in aligning AR images. This precision is vital for navigating complex sinus structures and antitarget avoidance. The findings indicate that AR can significantly aid in the precise placement of instruments along the frontal sinus pathway, suggesting that surgical navigation with AR could reduce the risk of complications and enhance surgical safety (80).

Furthermore, a comprehensive review assessed the current state of surgical navigation technologies. The review highlights that while existing systems generally achieve a TRE of 1.5–2.0 mm, there is a considerable need to improve this precision to 1.0–1.5 mm or, ideally, to 0.5–1.0 mm. Reducing the TRE would improve surgical navigation, directly impacting the effectiveness of sinus and skull base surgeries. The authors call for innovations that could further enhance the accuracy of surgical navigation systems (57).

### Transcranial approaches

AR/VR/MxR/XR creates patient-specific cranial models for preoperative planning of interventions such as pterional, bicoronal, supraorbital, and subfrontal craniotomies. These models enhance surgical execution by optimizing the surgical positioning, approach selection, and craniotomy placement in transcranial approaches (Table 1).

3D modeling and AR/MxR/XR technologies may minimize morbidity, particularly in anterior skull base meningiomas. These tumors are close to critical structures like the ICA, anterior cerebral arteries, optic and other cranial nerves, and pituitary gland, making accurate resection imperative. In managing anterior cranial base tumors extending into critical neuroanatomical corridors, 3D modeling stands at the forefront of surgical innovation. These technologies are now highly utilized in cases involving neoplasms such as tuberculum sella, planum sphenoidale and olfactory groove meningiomas, and chondrosarcomas, where the risk of iatrogenic damage to the cranial nerves and adjacent structures is significant. The 3D reconstructions enable surgeons to delineate tumor margins with greater accuracy, thereby enhancing the ability to preserve neurological function while ensuring near or gross total resection. Each application of these technologies substantially improves patient outcomes in skull base neurosurgery. Moreover, the VR platform's pro's interactive features are highly beneficial in transcranial routes. They serve as a comprehensive view of the surgical field that overcomes the limitations of traditional 2D imaging. This technological leap marks a milestone in the evolution of neurosurgical protocols and could set a new standard for the surgical management of intracranial pathologies (81).

In a video article, the authors used AR to perform a mini-pterional craniotomy and extradural clinoidectomy on a 69-year-old patient with clinoid meningioma. This approach leveraged a 3D VR model for surgical planning, which was then projected into the navigation-tracked microscope's eyepiece during surgery, enabling real-time AR guidance. This technique facilitated the surgical performance, allowing for an optimal surgical opening with a TRE better than 1.5 mm. This novel use of AR in surgery underscores more accurate and safer surgical interventions and pre-validated surgical plans directly onto the patient's anatomy during surgery (82).

A recent study investigated the utility of 3D VR in preoperative planning for patients with anterior skull base meningiomas. A retrospective analysis of 30 patients revealed that VR-based reconstructions can significantly improve the detection of tumor-related anatomical structures (85% accuracy with VR vs. 74% with conventional imaging, *p* = 0.002). The VR modality may alter neurosurgeons’ decisions regarding head positioning during surgery (37% lateral rotation recommended with VR compared to 27% with standard imaging, *p* = 0.009) and influence the choice of surgical approach (36% preferring pterional or extended pterional approaches with VR guidance, *p* = 0.03). The angles and approaches recommended with VR were determined to provide better exposure of critical anatomical skull base structures, thereby facilitating safer and more effective tumor resection. This angle allowed surgeons to optimize their view and access to the tumor, reducing the need for excessive brain retraction and minimizing potential damage to surrounding tissues. Although similar adjustments can be made with traditional methods, the VR system's ability to simulate these angles preoperatively gives surgeons a clearer understanding of the optimal positioning, which could provide more precise surgical interventions (6). However, it is essential to highlight that these findings were based on retrospective data and surgeon-based evaluations. The clinical outcomes, such as postoperative recovery and complication rates, were not directly assessed. Future studies should focus on objective outcome measures to confirm the clinical advantages of VR.

One of the initial studies validates the utility of three-dimensional multi-fusion volumetric imaging (3D MFVI) in preoperative simulations for skull base tumors. It integrates CT, MRI, and DSA to form 3D reconstructions that vividly depict the tumor's spatial relationships. This approach not only aids in planning the open neurosurgical pathway but also anticipates and navigates potential intraoperative challenges (83).

Central to their methodology is employing image-analyzing software that combines data from various imaging modalities through volume rendering and image fusion, facilitating a detailed visualization of the microsurgical anatomy. Such visualization supports surgeons in evaluating different surgical approaches, thereby enhancing the safety and efficacy of tumor resection strategies (72). The fundamental study demonstrated that 3D MFVI can predict tumor resectability and identify critical anatomical markers that influence the choice of surgical approach, whether via anterior, middle, or posterior skull base routes. The study evaluated 21 skull base tumors.

(SBTs) in 20 patients, including acoustic neurinomas (8), jugular neurinomas (3), meningiomas (4, with one being olfactory groove meningioma), chordomas (3), and others like facial and hypoglossal neurinomas and a dermoid cyst. The results supported the effectiveness of 3D MFVI in surgical planning. The study concludes that 3D MFVI is valuable in visualizing microsurgical anatomy, can improve surgical approach and precision for SBTs, and can be an educational tool for training (73).

## Middle skull base surgery

**Figure 2:**
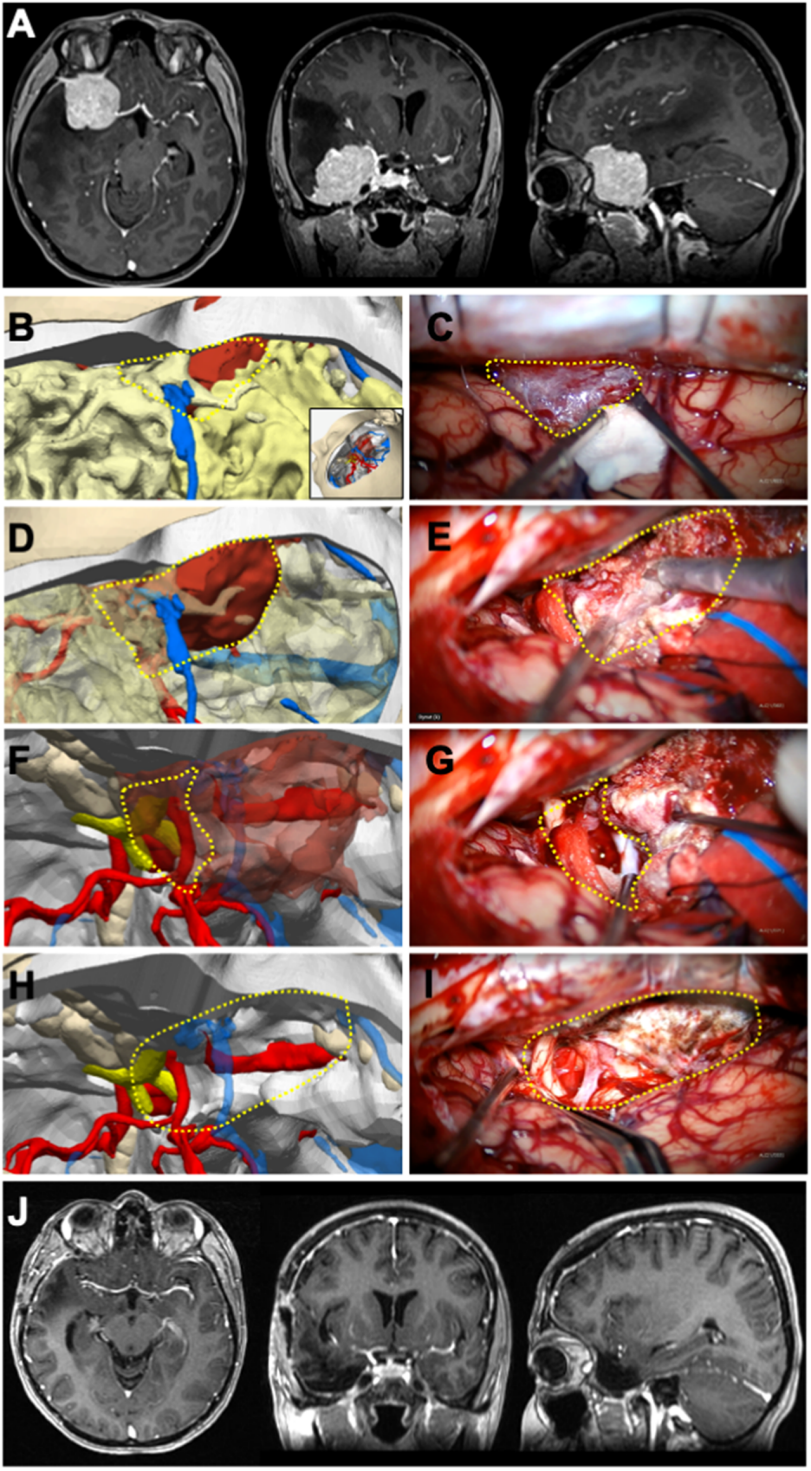
Illustrative case 2—sphenoid wing meningioma in a 34-year-Old female patient. (**A**) Preoperative axial, coronal, and sagittal MRIs enhanced with gadolinium contrast revealed a right sphenoid wing meningioma. (**B**) The 3D digital model was positioned surgically, a pterional craniotomy was performed, and part of the mass became visible at the craniotomy margin. (**C**) The portion of the tumor that is typically obscured by bone but was pulled into the surgical field is marked with a dashed yellow triangle. The venous structure relationship with the tumor, as seen under the microscope, closely resembled that of the model. (**D**) The segment of the tumor within the Sylvian fissure was rendered semi-permeable on the 3D model to predict its relationship with the cisternal neurovascular structures. (**E**) The tumor dissection within the Sylvian fissure was carried out microscopically, guided by the plan established on the model. (**G**) Retracting the anterior pole of the tumor microscopically, (**F**) as depicted in the 3D model, revealed the right optic nerve and right internal carotid artery (ICA). The retracted segment is indicated with a dashed yellow circle. (**I**) Following total excision, both optic nerves and the right ICA were visible at the surgical site, correlating with the 3D model. (**J**) Postoperative axial, coronal, and sagittal MRIs enhanced with gadolinium contrast confirmed the achievement of gross total excision.

The anterior boundary of the middle skull base and fossa is formed by the larger wings of the sphenoid bone, while the posterior limit is made up of the clivus. Horizontally, it intersects with the squamous part of the temporal bone and the anteroinferior part of the parietal bone. The middle fossa contains anatomical structures, including the sella turcica, which harbors the pituitary gland. The sella turcica is situated between the anterior and posterior clinoid processes. Additionally, there are important foramina, such as the foramen rotundum, ovale, and spinosum, which allow the passage of vital cranial nerves and vessels. The petrous section of the temporal bone serves as the posterior and medial boundary, enclosing the trigeminal ganglion in Meckel's cave, a crucial region above the foramen lacerum. This region's pathologies include complex tumors such as sphenoid wing meningioma (Figure 2), cavernous sinus pathologies, and sellar/parasellar pathologies, and studies have shown that 3D modelization and AR/VR technologies can be highly helpful before and during the surgeries as demonstrated in Table 1. The middle skull base's structural complexity is important for supporting the brain and presents specific complications during surgical procedures because of its proximity to vital neurovascular structures. The neurovascular complexities of the middle cranial fossa are segmented to create a realistic surgical environment. These segmented 3D models render bones with variable transparency, thus unveiling surgical anatomy layers. Such innovations facilitate surgical dissections and enhance not only neurosurgical planning and execution but also education and training (60).

### Endoscopic approaches

AR technologies have addressed complex pathologies such as parasellar meningiomas, pituitary adenomas, and craniopharyngiomas. By integrating 3D colored objects into the real-time surgical view, surgeons can navigate with a depth of the middle cranial skull base through the endoscopic pathways (64). These models are crucial before and during the surgeries when the surgical approach requires manipulating the bony structures and neurovascular components that are densely concentrated in this region. The real-time AR visualizations enhance the surgeon's perspective and aid in preoperative planning. This allows for a more strategic approach in real time to excising lesions while minimizing operative morbidity (62).

The integration of AR with endoscopic neuronavigation systems, such as the Scopis Navigation System, has started to be used in pediatric neurosurgery, particularly for complex middle skull base pathologies such as craniopharyngiomas, Rathke cleft cysts, and pituitary adenomas. This technology enables the superimposition of preoperative 2D imaging scans directly into the field of surgery during endoscopic procedures (84). AR-assisted surgery helps surgeons by providing a real-time, augmented view of the lesion's boundaries and essential surrounding structures. In a pediatric study, the team presented AR-assisted neuronavigation for endoscopic surgery on midline skull base pathologies in pediatric patients. Over nine years of experience, 17 endoscopic AR-assisted procedures were performed on children with lesions in the sellar and/or parasellar region. The patients (mean = 14.5 years) presented with various diagnoses. The most common one was craniopharyngiomas, at 31.2%. AR navigation was beneficial for accurately targeting lesions and determining their intraoperative extent. Postoperative MRI confirmed radical removal in 65% of oncological cases, with a mean follow-up period of 89 months. There were no fatalities, and only two cases of cerebrospinal fluid fistulas and a secondary abscess required additional surgeries. The study showed that AR offers information directly from the surgeon's field of view, which is valuable given the anatomical variability and rare pathologies in the pediatric population (85).

In the study by Goto et al., the authors introduced a novel AR navigation system incorporating three-dimensional computer graphic (3DCG) modeling for endoscopic transsphenoidal surgery, targeting sellar and parasellar tumors. This approach was developed to address the challenge of accurately identifying tumor locations and their relationships to surrounding structures, which are often distorted due to the tumor's presence. The system was evaluated across 15 patients, achieving an average usefulness score of 4.7 out of 5, indicating high effectiveness in surgical navigation. The AR system detailed 3DCG models from preoperative imaging onto real-time surgical views, offering surgeons a 3D understanding of the surgical field. Despite its advantages, the system's efficacy varied slightly among surgeons, especially for the depth perception of lesions. This emphasizes the importance of experience in interpreting AR visualizations. The study underscores the importance of the AR system for middle skull base tumors (86).

### Transcranial approaches

Integrating AR with 3D printing for patient consultations uses advanced software like Hyperspaces®. In the case of complex skull base cholesteatomas, CAD software translates CT scans into a series of 3D models. These models are then linked within AR platforms and accessed via mobile devices. Models bring patient-specific clinical pathologies into a format that is understandable for patients and physicians at minimal cost (87). Constructing interactive 3D models for the skull base uses software such as Maya to create a detailed and manipulable virtual anatomic landscape. This computer-generated model enables uninterrupted observation and study, promoting better surgical foresight and patient-specific operative planning (88).

The MxR platform was implemented in a research study utilizing the Microsoft HoloLens® to visually represent the anatomical structure of the middle skull base for lateral skull base approaches. This technology creates 3D holograms using CT images of cadaver heads and temporal bones. The process incorporated a semiautomatic and manual segmentation to construct 3D models. These models were then integrated into an MxR environment, developed via C# programming, enabling the display of dynamic 3D holograms on the HoloLens headset. This platform allowed users to interact with the virtual images through gaze, voice, and gesture commands. The accuracy assessment measured the average TRE of 5.76 mm ± 0.54 (63).

Creating the 3D models to aid skull base surgery education utilizes comprehensive cadaveric dissections alongside software for generating virtual replicas. These replicas allow for an improved understanding of neuroanatomical relationships and surgical approach selection when used alongside 2D radiographic imaging. Such resources are adjuncts to preoperative planning and critical in facilitating trainee evaluation (45). Software like Mimics converts imaging data into 3D reconstructions, which are then printed to simulate the surgical procedure. The study implemented 3D printing to develop individualized cranial nerve models for skull base tumor surgery. This innovative approach was applied to three patients: two with sellar tumors and one with an acoustic neuroma. The 3D-printed models encompassed detailed representations of the skull, brain tissue, blood vessels, cranial nerves, tumors, and other significant structures. The models facilitated surgical simulation, allowing surgeons to previsualize and strategize the surgical removal of the tumor while preserving vital cranial nerves. The process involved creating 3D reconstructions from patients’ preoperative imaging data, including CT and MRI scans, using specific imaging sequences and diffusion tensor imaging-based fiber tracking. The study's findings suggest that 3D-printed cranial nerve models significantly aid in the preoperative planning of skull base surgeries, helping to minimize cranial nerve damage (68).

In a study that Pojskić et al. conducted involving 39 patients undergoing surgery, especially for anterior and middle skull base meningiomas, AR was used with iCT for navigation. Most cases, specifically 26 (66.6%), achieved gross total resection. The study confirmed high registration accuracy with an average TRE of 0.82 ± 0.37 mm. The AR technology, integrated into the surgical microscope, significantly improved surgical precision. It enabled better visualization of neurovascular structures without any reported injuries. This approach underscores the potential of AR as a valuable tool in complex skull base surgeries, facilitating safer and more effective tumor resections (89).

VR augmentation employs software capable of rendering high-resolution 3D images. These models offer a virtual operating field, enhancing the surgeon's capabilities with detailed visualizations of the surgical scenario. Rosahl et al. evaluated VR augmentation for its utility in skull base surgery. Their study included data from 110 patients with various skull base pathologies, including sellar and parasellar tumors, lesions in the temporal bone, acoustic neuromas, various other cerebellopontine angle tumors, epidermoids, brainstem lesions, glomus tumors, craniocervical meningiomas. The primary imaging data, encompassing MRI, CT, and CT angiography, facilitated the creation of a virtual operating field (VOF) with translucent surface modulation and an optional “fly-through” video mode. This innovative approach aimed to enhance image guidance in skull base procedures. The VOF was utilized with an infrared-based image guidance system, allowing real-time comparison with the patient's anatomy during surgery (74).

While integrating AR/VR/MxR/XR technologies into transcranial approaches has shown promising results, it is essential to note that the current level of evidence is primarily based on retrospective case series. Comparative studies between these advanced technologies and conventional approaches are limited. Prospective case-control studies are necessary to establish these technologies’ clinical efficacy and safety. These studies should compare outcomes such as surgical precision, complication rates, and patient recovery times between traditional methods and those enhanced with AR/VR/MxR/XR.

## Posterior skull base surgery

The posterior skull base represents a challenging anatomical area, demanding surgical precision and extensive knowledge of its intricate structures. Recent advancements in surgical approaches, notably the integration of AR technologies, have significantly contributed to enhancing operative outcomes and reducing perioperative morbidity, as depicted in Table 1 (54). The application of AR in posterolateral skull base surgery is particularly promising. The technology allows for a fusion of virtual information with the surgical environment. It enables subsurface anatomy and pathology to be superimposed onto the surgeon's real-time surgical view during procedures such as vestibular schwannomas or petroclival meningiomas (90, 91). This is highly advantageous when navigating the anatomical complexities near the clivus and cerebellopontine angle. The differentiation between tumor tissue and neurovascular structures like facial nerves is paramount (53, 65).

## Endoscopic approaches

The use of 3D models in endoscopic approaches to posterior cranial base lesions is limited in the current literature. In a study, a team utilized patient-specific 3D printing, AR, and surgical navigation to facilitate the transcanal endoscopic approach to the petrous apex. The process began with manual segmentation of CT images to generate 3D models. Then, the study explored the use of AR for virtual preoperative planning. This virtual exploration allowed surgeons to comprehend anatomy and decide surgical strategies. A 3D-printed physical model of the patient's temporal bone, incorporating anatomical landmarks and the cyst, was created to simulate the surgical procedure. This allowed for tactile and spatial understanding of the petrous bone, middle and posterior skull base. Navigation technology was employed during the simulation and surgery. This integrated approach, combining tactile, visual, and virtual parameters, provided a better understanding of the surgical field, potentially improving patient outcomes (11). The MxR simulator combines VR software like Unity with physical 3D-printed models can create a hybrid training environment. The physical interaction with the 3D-printed models, tracked by systems such as the HTC Vive®, offers realistic surgical simulation for training purposes. It is a cost-effective and accessible solution for education and training surgical techniques with surgical planning (75).

The study conducted by Cabrilo et al. utilized AR-assisted neuronavigation to improve the accuracy of endoscopic skull base surgery in a patient with recurrent clivus chordoma. The researchers utilized AR to display preoperatively segmented images of anatomical structures in the operating field. This enabled the imaging of the tumor and essential structures, including the carotid and vertebral arteries, in real time during surgery. The technology allowed for the modification of image transparency and superimposition depth to align with the surgical focus, greatly assisting surgical navigation. The study emphasized the benefits of incorporating AR into endoscopic techniques for posterior skull base surgeries, improving safety and accuracy (71).

## Transcranial approaches

A recent study evaluated the effectiveness of a new mobile AR navigation system in guiding retrosigmoid craniotomy procedures, specifically for the precise anatomical point of the transverse-sigmoid sinus junction. The study included patients who underwent surgeries for conditions such as acoustic neuroma, trigeminal neuralgia, and hemifacial spasm. Results emphasized the system's accuracy, with a matching error averaging 2.88 ± 0.69 mm and the positioning time required being 279.71 ± 27.29 s on average. The system successfully identified and exposed the inner edge of the junction in all cases. These findings suggest that the system provides a reliable and cost-effective option for enhancing surgical efficiency in posterior skull base surgery through accurate positioning (66).

Recent studies showcased AR's integration with standard neuronavigation equipment and microscopes, showing that preoperative MRI and CT data can be effectively utilized intraoperatively to guide the resection of lesions like chordomas. These technological enhancements give surgeons a more comprehensive understanding of the deep-seated pathologies within the posterior skull base (71). In a study involving nearly 40 patients undergoing posterior skull base surgery over two years, AR technology was employed to improve surgical preparation and approach phases. Utilizing systems like the BrainLab Curve™, Surgical Theater, and a Zeiss OPMI PENTERO® 900 microscope, critical structures and points of interest were projected onto the surgical field. This AR application allowed for a delicate surgical approach, optimizing skin incision and maximizing craniotomy effectiveness by visualizing anatomical features such as the dural venous sinuses. Creating a 3D “fly-through,” alongside preoperative imaging, also facilitated a deeper understanding of the pathology. The study suggests that AR can significantly aid preoperative planning and the initial phases of skull base surgeries (67). AR's ability to project a detailed, layered image onto the surgeon's field of view significantly improves the precision of navigation around the brainstem and other vital structures (62, 66). Such technologies have facilitated mental 3D model building, leading to better situational awareness and a lower likelihood of morbidity (65). Visualizing dural venous sinuses through AR systems facilitates optimizing the skin incision and maximizing the craniotomy (92).

To enhance surgical training in selecting skull base approaches for posterior fossa tumors, a team developed open-source 3D models, focusing on seven cases identified from a skull base registry. These cases, chosen based on the feasibility of access through at least three posterior fossa craniotomies, were delicately segmented and modeled. The project created realistic 3D models for each primary operative approach and two alternatives available in a platform-neutral format for broad AR/VR and 3D printing applications. This initiative marks a significant advancement in surgical education, utilizing open-source principles to improve understanding of complex neuroanatomy and pathology (93).

The integration of various AR technologies and heads-up display systems have proven their worth in cerebellopontine angle surgeries. Despite the potential for cognitive overload due to a crowded visual field, careful management of the displayed information can mitigate such risks and avoid unintentional harassment (94). Moreover, the segmentation and labeling of critical structures facilitated by AR are vital in accurately navigating surgeries within the posterior cranial fossa. In addition to aiding in surgical planning, AR interfaces help reduce cognitive load and operative time, as reported in studies involving all types of skull base procedures (58, 69, 95).

**Figure 3:**
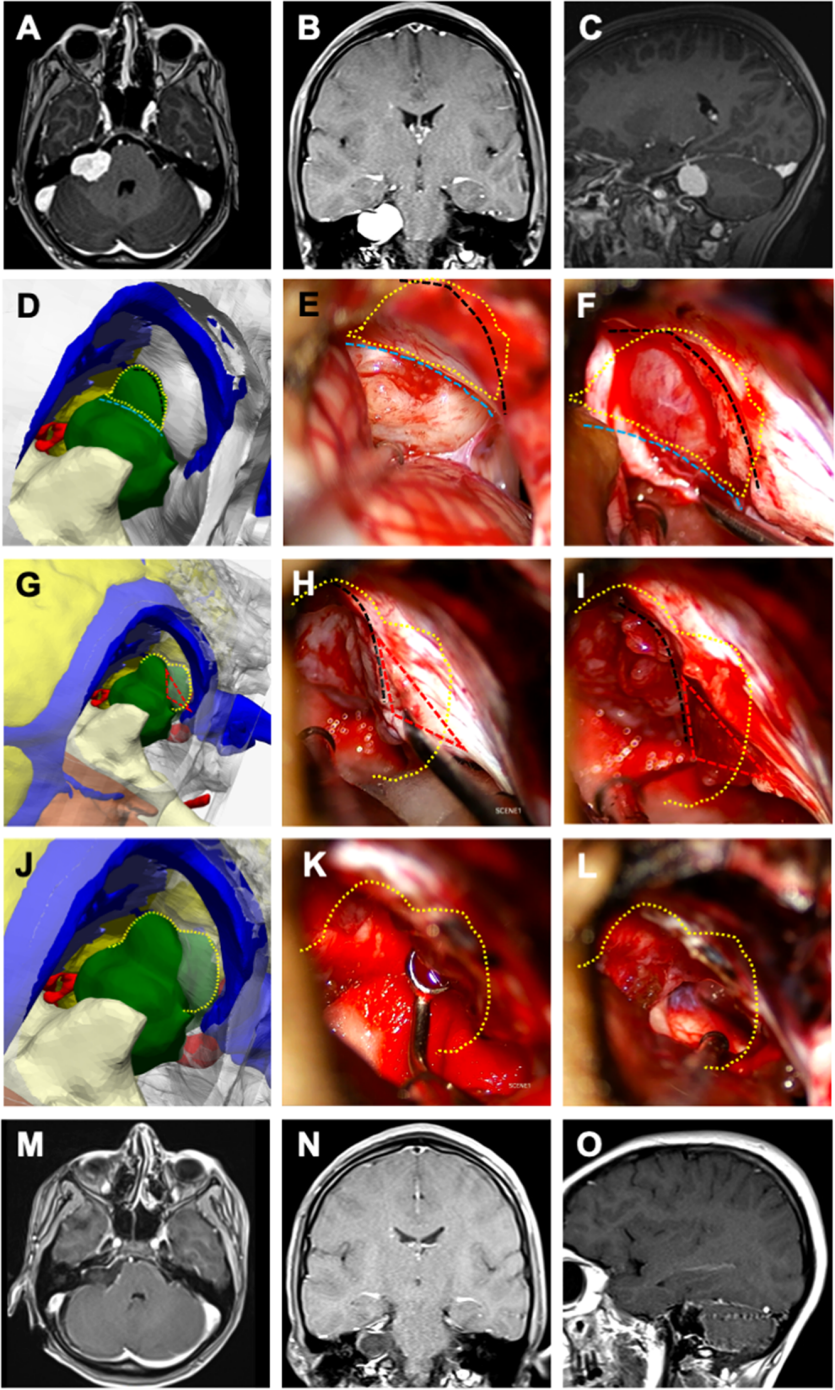
Illustrative case 3—vestibular schwannoma in a 13-year-Old female patient. A right vestibular schwannoma was evident on T1-weighted preoperative (**A**) axial, (**B**) coronal, and (**C**) sagittal MRIs with gadolinium contrast. (**D**) Surgical position was given to the 3D digital model (inset), right retrosigmoid craniotomy was performed, cerebellum tissue was removed, the tumor was colored green, and the transverse-sigmoid sinus was colored blue. The cisternal part of the tumor was exposed; the intradural portion of the tumor was demarcated with a dashed yellow circle, and the dura mater border was marked with a dashed cyan line. (**E**) In the microscope image, the cisternal part of the tumor corresponded closely with the model; the intradural projection of the tumor was outlined with a dashed yellow circle, the dural border with a dashed cyan line, and the planned dural incision with a dashed black line. (**F**) Following the excision of the cisternal segment, the dura mater was incised along the dashed black line to reveal the tumor component microscopically. (**G**) On the 3D model, the dashed yellow line indicates the lateral border of the tumor within the internal acoustic canal (IAC). The planned excision of the IAC roof is marked with a red triangle. (**H**) Microscopically, after the intradural segment was removed, the dashed black line delineates the bone edge, and (**I**) the bony roof marked with the red triangle was excised. (**J**–**L**) The tumor component within the canal was removed using a curette. Postoperative (**M**) axial, (**N**) coronal, and (**O**) sagittal MRIs with gadolinium contrast showed that a gross total excision was achieved.

In conclusion, posterior cranial base surgery continues to benefit from technological advancements, with AR/VR/MxR/XR as a significant innovation. These technologies aid in preoperative planning and execution and enhance the surgeon's understanding of complex anatomy, contributing to more effective surgical outcomes (Figure 3). As we continue to embrace these advancements, it is crucial to conduct further studies with more extensive series to substantiate the anecdotal evidence and refine the application of AR in clinical practice.

## Education and training

Integrating 3D visualization and reality technologies into neurosurgical training programs has shown significant potential in enhancing the educational experience for residents (96). These advanced tools provide immersive and interactive learning environments, offering several advantages for developing theoretical knowledge and surgical skills such as craniotomy planning (97). Simulation-based training using AR, VR, MxR, and XR technologies allows students or residents to engage in realistic surgical scenarios without the risks associated with actual procedures. VR platforms enable residents to practice complex skull base surgeries in a controlled and safe environment, improving their visuospatial skills and familiarity with intricate anatomical structures.

A recent study by Lai et al. validated a VR simulation for the middle cranial skull base approach using CardinalSim software. The study involved 20 trainees from neurosurgery, otolaryngology, and head and neck surgery. The results showed significant improvements in postsimulation test scores compared to presimulation scores (*P* < 0.001). Trainees demonstrated statistically significant improvements in the time to complete dissections (*P* < .001), internal auditory canal skeletonization (*P* < .001), completeness of the anterior petrosectomy (*P* < .001), and a reduced number of injuries to critical structures (*P* = .001). These findings underscore the effectiveness of VR in enhancing anatomical understanding and surgical skills, providing a valuable supplement to cadaveric dissections and live surgeries (98).

Munawar et al. also introduced the Fully Immersive Virtual Reality System (FIVRS) for skull-base surgery, which combines advanced surgical simulation software with high-fidelity hardware. FIVRS allows surgeons to follow clinical workflows inside a VR environment and uses advanced rendering designs and drilling algorithms for realistic bone ablation. The system also records extensive multi-modal data for post-analysis, including eye gaze, motion, force, and video of the surgery. Preliminary data from a user study involving surgeons of various expertise levels indicated that FIVRS could differentiate between participants’ skill levels, promising future research on automatic skill assessment. Informal feedback from study participants about the system's intuitiveness and immersiveness was positive, highlighting its potential for surgical training and skill assessment (99).

Moreover, Campisi et al. conducted a systematic review of the role of AR neuronavigation in transsphenoidal surgery. The review emphasized that AR enhances surgical education, training, preoperative planning, and intraoperative navigation. AR helps minimize the anatomical challenges associated with traditional endoscopic or microscopic surgeries, improving the safety and accuracy of skull base interventions. This systematic review highlights AR's potential to significantly improve surgical outcomes by providing real-time guidance and enhancing the surgeon's or residents’ spatial awareness during procedures (100).

These studies demonstrate that reality technologies provide valuable supplements to traditional training methods such as cadaveric dissections and live surgeries. By allowing residents to practice in a risk-free, controlled environment. These tools improve their visuospatial skills, enhance their understanding of complex anatomical structures, and prepare them for actual surgical scenarios.

Recent advancements in photogrammetry and VR have significantly improved the realism and accuracy of these anatomical models (12, 13). Corvino et al. demonstrated the effectiveness of photorealistic 3D model reconstruction of the sellar region for neurosurgical anatomy training. Using photogrammetry methods on four head specimens, the researchers created high-fidelity models replicating bony structures with high realism and accuracy. The interactive nature of these models allows for a 360° self-guided tour, providing a realistic spatial perception of anatomical relationships and depths. This interactive exploration aids residents in learning the sellar region's complex anatomy from transcranial and endonasal perspectives, enhancing their understanding and preparedness for actual surgical procedures (101).

Using 3D models and AR/VR platforms facilitates collaborative learning among residents and between residents and instructors. These tools enable interactive discussions and assessments, allowing instructors to highlight specific anatomical features and surgical steps. Additionally, simulation-based assessments can objectively evaluate residents’ skills and progress, providing targeted feedback and identifying areas for improvement.

Adopting these technologies in neurosurgical education offers a transformative approach to resident training, especially in more complex areas like skull base surgery. By providing immersive, interactive, and detailed learning experiences, these tools enhance the overall educational process, better preparing residents for the complexities of skull base surgery. As these technologies evolve, their integration into neurosurgical training programs is expected to become more widespread, ultimately leading to improved surgical outcomes and patient safety.

## Limitations, possible solutions, and future directions

Our study emphasizes the transformative potential of 3D visualization and reality technologies such as AR/VR systems in neurosurgical planning and intraoperative navigation in skull base surgeries. Over the years, these technologies have significantly improved surgical precision by providing patient-specific anatomical visualizations. For example, the reduction in TRE to 0.83 ± 0.44 mm with iCT-based automatic registration compared to 2.33 ± 1.30 mm with manual fiducial-based registration exemplifies the advancements in precision and reliability of these technologies (62).

However, several limitations need to be addressed. One major issue is the “crowding of objects” in these applications, where excessive objects can cause cognitive overload for neurosurgeons and reality algorithms. This can be mitigated by customizing reality algorithm interfaces to prioritize and highlight essential information, reduce cognitive overload, and improve focus during surgeries. Future research could focus on developing more intuitive user interfaces that allow for customizable displays according to the surgeon's preferences and the specific requirements of the surgery.

The weight of head-mounted devices (HMDs) can pose a significant issue regarding their use. Especially during lengthy skull base procedures, HMDs may lead to discomfort and fatigue for neurosurgeons, potentially affecting performance, precision, and patient outcomes. Addressing this limitation requires ergonomic advancements to create lighter and more comfortable HMDs. Additionally, exploring alternative display methods, such as lightweight glasses or integrated operating room displays, might alleviate some discomfort associated with HMDs (102, 103).

High-quality AR devices are not universally available. Their accessibility is limited by the resources of the healthcare institution, which has prevented widespread adoption in various medical settings. The initial investment and maintenance costs for advanced AR systems are substantial, creating a financial barrier for many hospitals. High-quality AR devices range from $3,000 to $10,000, while VR systems may cost between $1,000 and $5,000. Additionally, segmentation software licenses can cost upwards of $5,000–$20,000 annually. The time required to generate accurate models can vary from a few hours to several days, depending on the complexity of the case and the proficiency of the surgeon and the computer engineer. Future directions should consider developing cost-effective solutions and economic models to support integrating these technologies into routine clinical practice. This might include collaborative funding models, government grants, or partnerships with technology developers to reduce the financial burden on healthcare institutions.

Current MR scanning technology limits precise anatomical segmentation due to its resolution constraints. The insufficiencies in segmentation accuracy hinder detailed anatomical visualization. However, advancements in high-resolution imaging modalities and rendering technologies supported by AI are expected to overcome these challenges. Future developments should focus on creating open-access, high-resolution data sets to facilitate more accurate segmentations and enhance the utility of 3D models in surgical planning.

Integrating HMDs with existing surgical navigation systems can be complex, requiring sophisticated software and hardware alignment to ensure accurate and synchronized AR overlays. Technical issues such as calibration errors, latency, and software glitches can disrupt the surgical workflow, reducing the technology's reliability during critical moments (104).

It is essential to acknowledge the phenomenon of brain shift that can occur during transcranial approaches, particularly in large tumor resections. Brain shift refers to the displacement of brain tissues during surgery, which can progressively reduce the accuracy of navigation systems and overlays provided by reality technologies. As the operation proceeds, the preoperative imaging data may no longer accurately represent the intraoperative anatomy, leading to potential discrepancies. This can create a false sense of security and pose significant risks when coupled with intention bias. Ragnhildstveit et al. highlighted the issue of brain shift in glioma surgeries, noting that AR can help disclose and compensate for intraoperative brain shift. Still, the effectiveness varies with the accuracy of the registration methods used. For instance, they reported that the TRE for AR systems varied significantly, with values ranging from 0.90 mm to 8.55 mm depending on the specific technique and application. Continuous intraoperative imaging and regular recalibration of navigation systems are crucial to mitigate these risks and ensure the highest level of surgical precision and patient safety. The study also emphasizes the need for consistency in AR workflows and the development of standardized measures to evaluate the accuracy and clinical utility of AR systems (105).

Continuous technological advancements and rigorous testing are crucial to improving these systems’ robustness and reliability. Collaboration with software developers to enhance compatibility and reduce latency is essential (106). Moreover, with ongoing progress in AI and machine learning, significant enhancements in automating and perfecting the procedure of generating 3D models are possible. These algorithms will result in increased efficiency and instant implementation (107).

The effective use of AR systems also necessitates adequate training and familiarity, with surgeons needing extensive training to seamlessly integrate these technologies into their workflow. Standardized training programs and continuous education are vital to help overcome the learning curve associated with AR/VR systems. Establishing comprehensive training curricula and simulation-based practice sessions can ensure that surgeons are well-prepared to utilize these advanced tools effectively. Additionally, incorporating AR/VR training into neurosurgical residency programs could help future surgeons become proficient with these technologies from the outset of their careers.

Data management and security pose additional challenges, especially when handling large volumes of imaging data. Developing robust data management systems that ensure compliance with health information regulations is crucial to address these concerns. Secure data storage solutions and encryption methods can protect patient information, allowing seamless surgical planning and navigation access. Another critical limitation is the potential for attention bias, especially in MxR setups. Surgeons might become overly focused on digital overlays, risking the oversight of critical real-world anatomical details. Addressing this issue involves developing interfaces prioritizing essential information and mitigating cognitive overload.

While 3D printing offers the distinct advantage of creating tangible, patient-specific physical models that surgeons can manipulate, AR, VR, MxR, and XR technologies take surgical planning and execution to an entirely new level. These advanced visualization tools provide dynamic, interactive environments that can be integrated directly into the surgical setting. Unlike static 3D printed models, AR and MxR technologies can overlay digital information onto the patient in real time during surgery, enhancing the surgeon's spatial understanding of complex anatomical structures. This capability allows for real-time adjustments and immersive simulations, which aid in preoperative planning and significantly improve intraoperative navigation. By offering a comprehensive view combining physical and digital elements, these technologies can help reduce surgical complications and improve patient outcomes, ultimately pushing the boundaries of precision and safety in skull base neurosurgery.

Promoting collaboration among different sectors is crucial to fully leverage the advantages of 3D modeling and AR/VR technology in clinical practice. Recent advances in photogrammetry (31, 108), computer vision, and simulation technologies (12, 13) can enhance the immersive potential and utilization of personalized 3D neurosurgical models in education, research, and practice (35, 109). Hence, we anticipate the transformation of skull base neurosurgery by integrating advanced technologies and improved computational capabilities. This transformation will bring a new era of tailored surgical interventions in skull base surgery.

Integrating novel technologies into clinical practice should follow established frameworks, such as the IDEAL (Idea, Development, Exploration, Assessment, and Long-term Study) collaboration framework. According to this framework, the respective technologies discussed in this review are primarily in the exploration and assessment stage, with some preliminary evidence supporting their utility. However, comprehensive assessment through well-designed prospective studies is essential to move these technologies toward broader clinical adoption and long-term evaluation. Future research should improve cost-effectiveness, computational efficiency, and user-friendly interfaces to facilitate wider adoption. Finally, comprehensive training programs and seamless integration into existing surgical workflows are vital for maximizing the benefits of 3D models and AR/VR systems. By addressing these challenges, we can significantly enhance the precision, reliability, and accessibility of these technologies, leading to improved surgical outcomes and patient safety in skull base neurosurgery.

## Conclusion

Incorporating patient-specific 3D models into surgical planning signifies a fundamental change in how complicated surgical procedures are approached. These models enhance comprehension of complex anatomical connections and allow surgeons to practice and predict different surgical situations. When used in conjunction with AR/VR/MxR/XR settings, these models become highly effective tools for improving communication among the surgical team and patients while acting as exceptional instructional materials. Maximum effectiveness can be obtained in approach selection, patient positioning, craniotomy placement, anti-target avoidance, and spatial relationship of neurovascular structures. While these advancements herald a new era of precision in surgical planning and execution, it is essential to recognize the challenges associated with their implementation. It is crucial to develop more cost-effective solutions and financial models to support their integration into routine clinical practice. Higher-resolution and more accurate 3D visualization and reality technologies must also be created. Advances in computational power and algorithms for faster processing could help streamline this process. In order to properly utilize the advantages of these advancements in enhancing patient care, it is imperative to consistently improve the technology, efficiently control expenses, offer education, and perform research to overcome current limitations. To address the current limitations and enhance the integration of these technologies into clinical practice, future research should focus on conducting prospective case-control studies that provide high-level evidence on the comparative efficacy of AR/VR/MxR/XR-enhanced techniques vs. conventional approaches. Additionally, adherence to frameworks such as IDEAL will facilitate structured evaluation and integration, ensuring these innovations improve surgical outcomes effectively and safely.
